# Frequency of Gluten-Reactive T Cells in Active Celiac Lesions Estimated by Direct Cell Cloning

**DOI:** 10.3389/fimmu.2021.646163

**Published:** 2021-03-16

**Authors:** Shuo-Wang Qiao, Shiva Dahal-Koirala, Linn M. Eggesbø, Knut E. A. Lundin, Ludvig M. Sollid

**Affiliations:** ^1^ K.G. Jebsen Coeliac Disease Research Centre, Department of Immunology, University of Oslo, Oslo, Norway; ^2^ Department of Immunology, Oslo University Hospital, Oslo, Norway; ^3^ Department of Gastroenterology, Oslo University Hospital, Oslo, Norway

**Keywords:** celiac disease, gluten, T cells, epitope, HLA, direct cloning

## Abstract

Chronic inflammation of the small intestine in celiac disease is driven by activation of CD4+ T cells that recognize gluten peptides presented by disease-associated HLA-DQ molecules. We have performed direct cell cloning of duodenal biopsies from five untreated and one refractory celiac disease patients, and three non-celiac disease control subjects in order to assess, in an unbiased fashion, the frequency of gluten-reactive T cells in the disease-affected tissue as well as the antigen fine specificity of the responding T cells. From the biopsies of active disease lesions of five patients, 19 T-cell clones were found to be gluten-reactive out of total 1,379 clones tested. This gave an average of 1.4% (range 0.7% - 1.9%) of gluten-reactive T cells in lamina propria of active celiac lesions. Interestingly, also the patient with refractory celiac disease had gluten-reactive T cell clones in the lamina propria (5/273; 1.8%). In comparison, we found no gluten-reactive T cells in any of the total 984 T-cell clones screened from biopsies from three disease control donors. Around two thirds of the gluten-reactive clones were reactive to a panel of peptides representing known gluten T-cell epitopes, of which two thirds were reactive to the immunodominant DQ2.5-glia-α1/DQ2.5-glia-α2 and DQ2.5-glia-ω1/DQ2.5-glia-ω2 epitopes. This study shows that gluten-reactive T cells in the inflamed duodenal tissue are prevalent in the active disease lesion, and that many of these T cells are reactive to T-cell epitopes that are not yet characterized. Knowledge of the prevalence and epitope specificity of gluten-specific T cells is a prerequisite for therapeutic efforts that target disease-specific T cells in celiac disease.

## Introduction

Celiac disease is a chronic inflammatory disease of the small intestine elicited by T-cell mediated immune response to dietary gluten. Almost all patients express the HLA-DQ2.5, HLA-DQ2.2 or HLA-DQ8 allotypes with HLA-DQ2.5 being expressed by more than 90% of the patients. The disease-associated HLA-molecules present gluten to CD4+ T cells. Gluten is a complex family of proteins found in wheat. Currently, 27 distinct HLA-DQ2.5-restricted T-cell epitopes have been characterized from gluten and related proteins such as hordein (barley), secalin (rye) and avenin (oat) (1). CD4+ T cells reactive to these epitopes can only be found in the intestinal tissue of celiac disease patients, and not in controls including non-celiac gluten-intolerant patients (2, 3). All HLA-DQ2.5-restricted gluten T-cell epitopes contain one or several glutamic residues within the 9-mer core HLA-binding region. Native gluten has few glutamic acid residues, but contains over 35% glutamine residues (4). Certain glutamine residues in the native gluten can be post-translationally modified into glutamic acid residues by the action of transglutaminase 2 (TG2), in a reaction known as deamidation (5). The negatively charged glutamic acid residues increase the binding affinity of gluten peptides to HLA-DQ2.5 (6).

Among the multitude of HLA-DQ2.5-restricted gluten T-cell epitopes, only a few are known to be recognized by nearly all celiac disease patients. These immunodominant epitopes are the epitopes DQ2.5-glia-α1, DQ2.5-glia-α2, DQ2.5-glia-ω1, DQ2.5-glia-ω2, and DQ2.5-hor-3 (7, 8). T cells that recognize the other HLA-DQ2.5-restricted gluten epitopes are found occasionally in a minority of celiac disease patients. Despite decades of efforts in characterizing T-cell epitopes from gluten (reviewed in 1), there are still undiscovered T-cell epitopes in gluten, a fact manifested by T-cell clones that recognize deamidated peptic digest of gluten, but not any of the known epitope sequences. The proportion of gluten-reactive T cells that recognize hitherto uncharacterized gluten epitopes is largely unknown since few systematic efforts have been made in the past few years.

The fact that an unknown fraction of T cells from celiac disease patients recognize yet unknown gluten epitopes, impacts the estimation of the frequency of gluten-reactive T cells in the celiac intestinal tissue. Most studies use ELISPOT with overlapping peptides from a few well-characterized gluten proteins (8, 9) or tetramers presenting a limited number of the known gluten epitopes (10, 11) in their estimation of this frequency. Only one single study has reported the frequency of gluten-reactive T cells by direct cloning of T cells from the lamina propria of duodenal biopsies (10). This study, however, included only three treated patients, and two untreated patients. The average frequency of gluten-reactive T cells in this study was 1.0% (range 0.5% - 1.8%, four patients) in celiac lesion with Marsh 3, a histological score that indicates the most severe tissue damage. Yet other approaches such as cloning from polyclonal T-cell line can give some indication of the specificity distribution, including the proportion of cells that recognize unknown gluten epitopes. However, due to *in vitro* expansion of the polyclonal lines, this approach does not give the precursor frequency of gluten-reactive T cells in the celiac lesion.

In the current study, we have cloned T cells directly from the lamina propria of total nine subjects, of whom five were untreated patients with Marsh 3 lesion, one refractory celiac patient with Marsh 3, and three control subjects that do not have celiac disease. From the six celiac disease patients with Marsh 3 in the duodenum, 24 T-cell clones were found to be gluten-reactive of total 1,652 clones tested, giving an average frequency of 1.5% of gluten-reactive T cells. We also found that around one third of gluten-reactive T-cell clones from these patients only responded to deamidated gluten, but not to peptides containing any of the known HLA-DQ2.5-restricted gluten epitopes.

## Material and Methods

### Patients and Biopsies

Biopsies were taken as part of routine clinical follow-up or to investigate a suspected diagnosis of celiac disease. The regional committee for medical research ethics had approved the relevant protocols (REK 6544), and patients gave written consent before participating. One or two pieces of biopsies were taken from the descending duodenum for this study, and additional biopsies were taken from the duodenal bulb (bulbus duodeni) for diagnostic purposes. Patients received diagnosis based on the guidelines from European Society for the Study of Coeliac Disease (ESsCD) (12).

Duodenal biopsies were transported in sterile RPMI on ice. The epithelial layer was stripped off by two consecutive incubations with 5 ml PBS + 2% FCS + 2 mM EDTA, 10 min each in a rotating tube at 37°C. Single-cell suspension of the remaining lamina propria preparations was made by incubation with 10 ml PBS + 2% FCS + 1 mg/ml collagenase (Sigma) and 100 µg/ml DNase (Sigma), for 45 min at 37°C.

### Cloning and Expansion

T cells were cloned and expanded in culture medium (RPMI (Gibco) + 10% human serum + 10 mM 2-ME (M-6250, Sigma) + penicillin/streptomycin) supplemented with 20 U/ml IL-2 (R&D systems), 1 ng/ml IL-15 (R&D Systems) and 1 µg/ml phytohemagglutinin (PHA, Remel), in the presence of 0.8 – 1 mill/ml irradiated (30 Gy) mixed allogeneic peripheral blood mononuclear (PBMC) feeder cells from 2-3 donors. For the initial cloning, single-cell suspension of duodenal lamina propria was washed, counted and re-suspended in the expansion medium containing the abovementioned ingredients. Cells in 20 µl were distributed into each well of the Terasaki plates (Nunc). Lamina propria cells were seeded at three different concentrations: 30, 10 and 5 cells per well. Growth was assessed microscopically after 10 days. Probability for clonal growth was calculated from the percentage of proliferating wells for each seeding concentration based on assumptions of Poisson distribution with the follow formula: P_clonal_ = -λ(e^λ^/(1-e^λ^)) where λ = ln(1-observed frequency of growth). Proliferating T cells were transferred from Terasaki wells to flat-bottomed 96-wells containing 125 µl of the expansion medium containing freshly prepared PBMC feeder cells and cytokine as described above. Fresh culture medium containing IL-2 and IL-15 were added about every two days, and about half of the old medium was removed when necessary. Eight to 10 days after expansion in 96-wells, proliferating T cells were screened for reactivity to deamidated gluten antigen. T-cell clone (TCC) that showed proliferative response were further expanded in two 24-wells each, containing 1 ml expansion medium. These TCC were further tested for gluten specificity and screened with a panel of peptides containing different HLA-DQ2.5-restricted gluten epitopes (**Supplementary Table 1**).

### Screening by T-Cell Proliferation Assay

TCC were tested in proliferation assays where gluten or peptide antigens were presented by HLA-DQ2.5-expressing antigen-presenting cells. Gluten was prepared in-house from wheat flour and digested with chymotrypsin according to procedures described in (13). Gluten was subjected to TG2-mediated deamidation where 2 mg/ml gluten was incubated in 100 mM Tris + 2 mM Ca^2+^ with 50-100 µg/ml human recombinant TG2 for 2 h at 37°C. In each U-bottomed 96-well, 50,000 – 70,000 irradiated (75 Gy) HLA-DQ2.5 homozygous EBV-transformed B lymphoblastoid cells (CD114) were pre-incubated overnight at 37°C with native or deamidated wheat gluten (100 µg/ml unless otherwise stated) or synthetic peptides (10 µM unless otherwise stated) that contained HLA-DQ2.5-restricted gluten T-cell epitopes. On the following day, T cells were added and incubated for another 3 days. One µCi ^3^H-thymidine (Hartman Analytics) was added 16-20 h before harvest and counting on a scintillation counter. Proliferative response was measured as stimulatory index (SI) defined as counts per minute (CPM) with antigen of interest divided by CPM with PBS only. For the initial screening of proliferating T cells from Terasaki wells, each TCC was tested in single well of either PBS or TG2-treated gluten (Gluten-TG2). TCC with SI above 2.5 from this single-well screening were selected for further expansion and testing. In subsequent assays, each antigenic condition was tested in either duplicates or triplicates. Only TCC that showed proliferative response (SI>2.5) to Gluten-TG2 or gluten peptide antigens in the subsequent re-testing was deemed gluten-reactive and included in the analysis.

### Tetramer Staining and Flow Cytometry

Selected gluten-reactive TCCs were stained with FITC-CD8 (clone SK1, BD), PE-CD4 (clone SK3, BD), or corresponding isotype controls together with propidium iodide (BioLegend). TCC that were reactive to one of the four epitopes: DQ2.5-glia-α1, DQ2.5-glia-α2, DQ2.5-glia-ω1 or DQ2.5-glia-ω2, were stained with 10 ng/ml of PE- or APC- conjugated HLA-DQ2.5 tetramer presenting one of these four epitopes (11), for 40 min at room temperature. Samples were analyzed on a FACS Calibur II (BD) and data analyzed with FlowJo (BD).

## Results

### T-Cell Clones Can Be Successfully Established by Direct Cloning of Lamina Propria Cells

In order to assess the precursor frequency of gluten-reactive CD4+ T cells in the active celiac disease lesion, we generated T-cell clones directly from unsorted lamina propria preparations of duodenal biopsies from nine individuals. These were subjects that were referred to gastroenteroscopy examination for suspicion of celiac disease. Five patients received the diagnosis of celiac disease based on Marsh 3 changes in histological examination; three subjects had none or minimal histological changes and did not receive the diagnosis. The last subject had refractory celiac disease, where gross morphological changes (Marsh 3C) persisted despite gluten-free diet (**Table 1**).

**Table 1:** Patient characteristics and frequency of gluten-reactive T cells.

Patient	Gender	Age	HLA	Clinical status	IgA-TG2*(ref <3)	IgG-DGP*(ref <20)	Other clinical information	Histology*	Gluten-reactivity among T-cell clones (P_clonal_ > 70%)	
CD1334	F	62	DQ2.5	CONTROL	1.1	**26**		1	0/269^¶^	0.0%
CD1346	M	47	DQ2.5	CONTROL	**9**	10		1	0/370^¶^	0.0%
CD1350	F	56	DQ2.5	NCGS	0	0	2wks gluten challenge	0	0/345^¶^	0.0%
**Total control subjects**									0/984	0.0%
CD1329	F	25	DQ2.5	UCD	**5.8**	9		3B	5/286	1.7%
CD1335	F	54	DQ2.5	UCD	**23**	**57**		3B	2/271	0.7%
CD1336	F	49	DQ2.5	UCD	2.5	**41**	Graves’ disease	3B	3/284	1.1%
CD1344	M	35	DQ2.5	UCD	**41**	12	1^st^ relative with CD	3B	5/263	1.9%
CD1349	F	27	DQ2.5	UCD	<1	**100**	IgA deficiency	3C	4/275	1.5%
**Total UCD**									19/1379	1.4%
CD1348	F	56	DQ2.5	RCD	<1	10	CD diagnosis 2004	3C	5/273	1.8%

*Histology and serological results from the time point of sampling.

^¶^All wells tested are included.

NCGS, non-celiac gluten sensitivity; UCD, untreated celiac disease; RCD, refractory celiac disease.

Serology results above cut-off values are denoted in bold.

We successfully cultured T cells by directly seeding unsorted single-cell suspensions of lamina propria preparations from descending duodenal biopsies from all nine subjects, using an antigen-free cloning and expansion protocol. After 10 days *in vitro* culture in the presence of PHA, IL-2 and IL-15, we found wells containing growing T cells in frequencies that were directly correlated with the seeding concentration of unfractionated lamina propria cells (**Table 2**). In most cases, seeding 10 or 5 lamina propria cells per well resulted in the growth of T cells in less than 50% of the wells, which implied >70% likelihood that the T-cell populations in each well were clonal. For simplicity, we will refer to these cells as T-cell clones (TCC).

**Table 2:** Detailed cloning frequency.

	Terasaki seeding	Frequency of growth, cloning in Terasaki plates		P_clonal_*	Frequency of growth, expansion in 96-wells		Frequency gluten-specific of tested T cells		Average frequency P_clonal_ > 70%		Average frequency all tested	
**CD1334** (Marsh 1)	30/well	101/120	84%	35%	76/96	79%	0/45	0%			0/269	0%
	10/well	111/240	46%	**72%**	78/96	81%	0/78	0%	0/224	0%		
	5/well	211/1200	18%	**91%**	158/192	82%	0/146	0%				
**CD1346** (Marsh 1)	30/well	98/120	82%	38%	92/96	96%	0/92	0%			0/370	0%
	10/well	98/210	47%	**72%**	92/96	96%	0/92	0%	0/278	0%		
	5/well	205/780	26%	**86%**	186/192	97%	0/186	0%				
**CD1350** (Marsh 0)	30/well	117/180	65%	57%	99/103	96%	0/99	0%			0/345	0%
	10/well	99/300	33%	**81%**	84/96	88%	0/84	0%	0/246	0%		
	5/well	185/1200	15%	**92%**	162/185	88%	0/162	0%				
**CD1329** (Marsh 3B)	30/well	202/240	84%	35%	95/96	99%	4/95	4.2%			9/381	2.4%
	10/well	143/300	48%	**71%**	95/96	99%	1/95	1.1%	5/286	1.7%		
	5/well	216/780	28%	**85%**	191/192	99%	4/191	2.1%				
**CD1335** (Marsh 3B)	30/well	127/180	71%	51%	96/96	100%	2/96	2.1%			4/367	1.1%
	10/well	107/300	36%	**80%**	111/115	97%	1/111	0.9%	2/271	0.7%		
	5/well	176/1100	16%	**92%**	160/173	92%	1/160	0.6%				
**CD1336** (Marsh 3B)	30/well	121/170	71%	50%	95/96	99%	4/95	4.2%			7/379	1.8%
	10/well	113/360	31%	**82%**	94/96	98%	1/94	1.1%	3/284	1.1%		
	5/well	195/1000	20%	**90%**	190/192	99%	2/190	1.1%				
**CD1344** (Marsh 3B)	30/well	104/150	69%	52%	92/96	96%	0/92	0.0%			5/355	1.4%
	10/well	104/300	35%	**80%**	91/96	95%	0/91	0.0%	5/263	1.9%		
	5/well	198/1075	18%	**90%**	172/192	90%	5/172	2.9%				
**CD1349** (Marsh 3C)	30/well	105/120	88%	30%	95/96	99%	2/95	2.1%			6/370	1.6%
	10/well	118/240	49%	**70%**	94/96	98%	2/94	2.1%	4/275	1.5%		
	5/well	227/840	27%	**85%**	181/192	94%	2/181	1.1%				
**CD1348** (Marsh 3C)	30/well	107/180	59%	62%	94/96	98%	2/94	2.1%			7/367	1.9%
	10/well	105/360	29%	**84%**	95/96	99%	1/95	1.1%	5/273	1.8%		
	5/well	193/1192	16%	**91%**	178/192	93%	4/178	2.2%				

*When P_clonal_, the probability that the growing wells is clonal, is larger than 70% (in bold), the wells are included in the calculations of frequency of gluten-specific T cells.

### Gluten-Reactive T Cells Were Only Found in Duodenal Biopsies From Active Celiac Lesions

From each of the nine subjects, 269-381 TCC were expanded and screened for proliferative response to gluten. In the three subjects with little or no histological changes in the duodenum, we found no gluten-reactive T cells in any of the total 984 TCC screened. In comparison, from the six celiac disease patients with Marsh 3 changes, including one refractory CD patient, 38 TCC, or 1.7%, were gluten-reactive out of total 2,219 clones tested.

Since growth was detected frequently at the top seeding concentration of 30 lamina propria cells per Terasaki well, there is a high likelihood that many of these wells contained cells that originated from two or more seeded cells and thus were not truly clonal. As a consequence, the frequency of gluten-reactive T cells could thus be slightly over-estimated due to oligoclonal growth in some wells. To correct for this, we counted only proliferating T cells that were more than 70% likely to be clonal in the calculation of the frequency of gluten-reactive T cells. Among TCC with P_clonal_ > 70%, we found 19 TCC that were gluten-reactive out of total 1,379 clones tested from the five untreated celiac disease patients with Marsh 3 changes in the duodenum (**Table 1**). This gave an average of 1.4% (range 0.7% - 1.9%) of T cells in lamina propria of active celiac lesions that were gluten-reactive. In addition, in the refractory celiac disease patient who had Marsh 3C changes in the duodenum, we found five gluten-reactive TCC of 273 tested. Thus, despite gluten-free diet, the frequency of gluten-reactive T cells in this patient was found to be as high as that found in untreated celiac disease patients that were exposed to dietary gluten antigen. When data from the refractory patient was grouped with the five Marsh 3 samples from untreated celiac patients, 24 gluten-reactive TCC were found in total 1,652 P_clonal_ > 70% TCC tested, giving an average of 1.5% (range 0.7% - 1.9%).

### Distribution of Gluten Epitope Specificity

**Figure 1:**
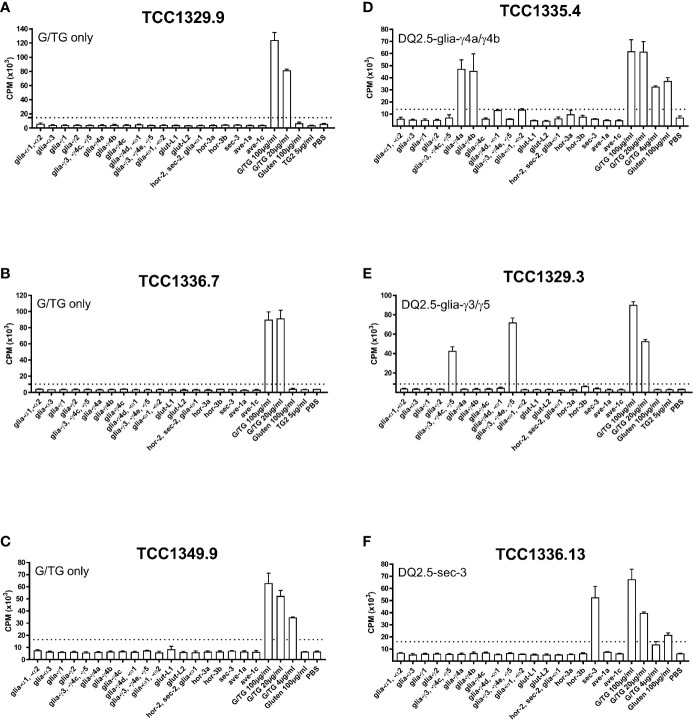
Proliferation of T-cell clones that were specific to TG2-treated gluten only, various DQ2.5-glia-γ epitopes, or DQ2.5-sec-3. **(A-C)** Three T-cell clones from three different patients that responded to TG2-treated gluten digest only. **(D)** A DQ2.5-glia-γ4a and DQ2.5-glia-γ4b reactive T-cell clone. **(E)** A DQ2.5-glia-γ3 and DQ2.5-glia-γ5 reactive T-cell clone. **(F)** A DQ2.5-glia-sec-3 reactive T-cell clone. T-cell proliferation measured by ^3^H-thymidine incorporation is visualized by CPM (counts per minute). Bars show average CPM response and standard error of the mean. Dotted line denotes the cut-off set at 2.5 times CPM with PBS. G/TG: TG2-treated gluten digest. All peptides were tested at 10 μM unless otherwise stated.

**Figure 2:**
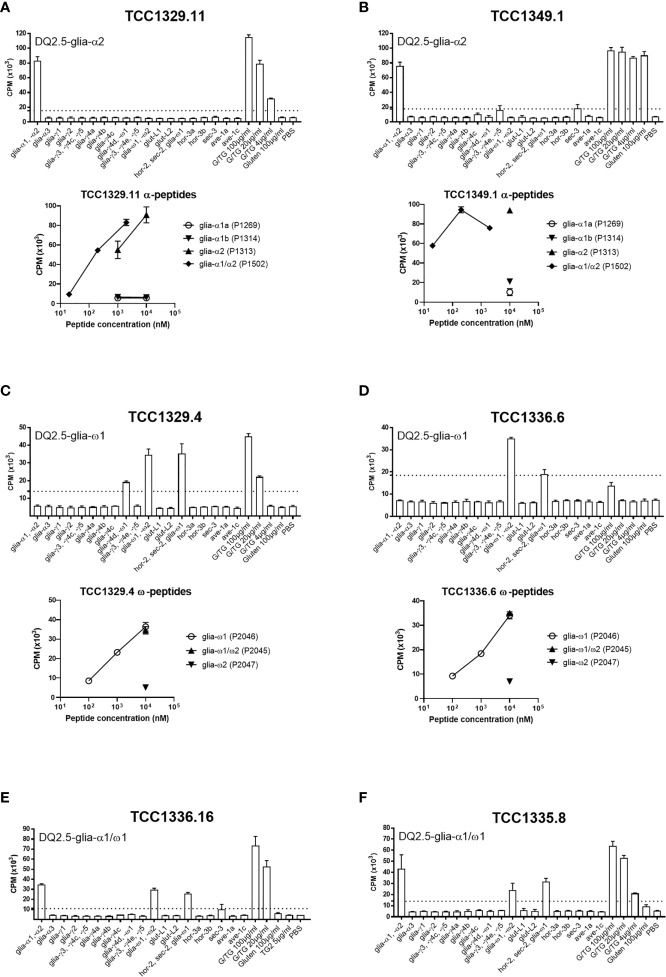
Proliferation of T-cell clones that were specific to immunodominant DQ2.5-glia-α and DQ2.5-glia-ω epitopes. **(A, B)** Two T-cell clones reactive to DQ2.5-glia-α2. **(C, D)** Two T-cell clones reactive to DQ2.5-glia-ω1. **(E, F)** Two T-cell clones reactive to both DQ2.5-glia-α1 and DQ2.5-glia-ω1. T-cell proliferation measured by ^3^H-thymidine incorporation is visualized by CPM (counts per minute). Bars show average CPM response and standard error of the mean. Dotted line denotes the cut-off set at 2.5 times CPM with PBS. G/TG: TG2-treated gluten digest. All peptides were tested at 10 μM unless otherwise stated.

All gluten-reactive T cells, including those generated with 30/well seeding in Terasaki wells, were screened against a panel of peptides that contained all known HLA-DQ2.5-restricted gluten epitopes (**Supplementary Table 1**), as well as 5 µg/ml recombinant TG2 in order to exclude any reactivity toward the TG2 component of TG2-treated gluten used in the initial screening. We found no TG2-reactivity in any of the T cells tested. Of the total 38 gluten-reactive TCC tested (**Table 3**), 12 TCC were reactive to deamidated gluten, but not to any of the peptides containing known gluten epitopes (**Figures 1A-C**). Of the 26 TCC where epitope specificity was ascertained, 17 were reactive to the immunodominant DQ2.5-glia-α1/DQ2.5-glia-α2 and DQ2.5-glia-ω1/DQ2.5-glia-ω2 epitopes (**Figure 2**) of which 15 were confirmed by specific staining with HLA-DQ2.5 tetramers presenting one of these four epitopes (**Supplementary Table 2** and **Supplementary Figure 2**), eight were reactive to various DQ2.5-glia-γ epitopes (**Figures 1D, E**) and one was reactive to the DQ2.5-sec-3 epitope (**Figure 1F**). Of note, there was no significant difference between the magnitude of response in terms of SI values, between gluten only, α-gliadin and/or ω-gliadin, γ-gliadin or secalin specific T cells (**Supplementary Table 2**).

**Table 3:** Summary of epitope specificities of gluten-reactive TCC.

	G/TG only	α1 or α2	ω1 or ω2	α1 & ω1	γ3 or γ5	γ4	sec-3	TOTAL
CD1329	4	2	2		1			9
CD1335		1		2		1		4
CD1336	2	1	2	1			1	7
CD1344	2	2				1		5
CD1349	3	1			2			6
CD1348	1	1	1	1	1	2		7
TOTAL	12	8	5	4	4	4	1	38

G/TG, TG2-deamidated gluten digest; α1: DQ2.5-glia-α1; α2: DQ2.5-glia-α2; ω1: DQ2.5-glia-ω1; ω2: DQ2.5-glia-ω2; γ3: DQ2.5-glia-γ3; γ4: DQ2.5-glia-γ4; γ5: DQ2.5-glia-γ5; sec-3: DQ2.5-glia-sec-3.

## Discussion

Gluten-reactive T cells are a hallmark of celiac disease. These cells recognize TG2-deamidated gluten presented on disease-associated HLA-DQ molecules and play pivotal roles in the pathogenesis of celiac disease. In the current study, we have generated T-cell clones directly from lamina propria tissues and found that all six samples taken from tissues in active disease contained gluten-reactive T cells. In average, 1.4% (range 0.7% - 1.9%) of T cells from inflamed celiac tissue were gluten-reactive, compared to zero reactive clones of total 984 TCCs generated from non-celiac tissues. We found that almost half of the gluten-reactive TCC were specific to the four immunodominant DQ2.5-glia-α1/DQ2.5-glia-α2 and DQ2.5-glia-ω1/DQ2.5-glia-ω2 epitopes, whereas combined, only a quarter of the TCC were specific to any of the other 23 HLA-DQ2.5 restricted gluten epitopes. More surprisingly, we found that one third of the gluten-reactive TCC were specific to some not yet discovered epitopes in gluten.

To our knowledge, only one previously published study has estimated the frequency of gluten-reactive T cells by direct cloning (10). Of the four Marsh 3 patients investigated in this study from 2013, the average frequency was 1.0% (0.5% - 1.8%) of all TCC tested. Our results are not statistically significantly different from this estimate. However, we could speculate that since the intestinal biopsies used in Bodd et al. (10) were mostly Marsh 3A, including from two subjects that were treated patients on gluten-free diet, less inflammation could be the cause of the somewhat lower frequency estimate found in that study.

Interestingly, a refractory celiac disease patient who had massive inflammation in the duodenum despite gluten-free diet, had gluten-reactive T cells with the same frequency as other untreated celiac disease patients with similar Marsh 3 changes. This finding is in accordance with Bodd et al. (10), where the frequency of gluten-reactive T cells estimated by direct cloning was correlated with the Marsh score, rather than with the gluten consumption status. Overall, our results further corroborate the notion that tissue injuries in celiac disease are driven by gluten-reactive T cells.

An important assumption for our estimate of gluten-reactive T cells based on *in vitro* cloning, is that all T cells have equal potential to grow under the *in vitro* culture conditions used. However, it is conceivable that recently *in vivo* activated gluten-reactive T cells could be less prone to proliferate *in vitro* due to activation-induced refractoriness. If that is the case, the frequency we have calculated would under-estimate the true frequency of gluten-reactive T cells. Similarly, our estimate would not include gluten-reactive cells that do not proliferate well under the *in vitro* culture and testing conditions used, including regulatory T cells. On the other hand, since 8%-29% of the TCC could in reality be oligoclonal as estimated by the growth in Terasaki wells, the frequency we have shown may slightly over-estimate the true frequency of gluten-reactive T cells. This last notion is supported by flow cytometry data where 7 of 37 gluten-specific TCC showed > 5% contamination with CD8+ T cells. Since the staining was performed after many rounds of expansion, the CD8+ T cells may have originated from unintended out-growth of feeder cells that is known to occur occasionally despite irradiation.

We have previously reported that gluten-reactive memory T cells persist for decades in celiac disease patient on gluten-free diet (11). A definite cure from the disease is therefore dependent on the eradication or ‘re-education’ of existing gluten-reactive memory T cells that were primed and formed during the active disease phase. It is clearly a shortcoming of the current study that we did not assess the frequency of gluten-reactive memory T cells in treated celiac disease patients on gluten-free diet. As a method for assessing the frequency of gluten-reactive T cells, direct cloning is labor-intensive. Other methods such as tetramer staining is much less labor-intensive assuming the availability of the key reagents. The current study and Bodd et al. (10) have both found that around half of all gluten-reactive T cells are specific to one of the four immunodominant DQ2.5-glia-α or DQ2.5-glia-ω epitopes. By using this knowledge, the total frequency of gluten-reactive T cells can thus be extrapolated from tetramer-staining data.

It is a weakness of this study that TG2-deamidated gluten was used as the sole antigen during the initial screening. It is therefore likely that T cells that recognize hordein, secalin and avenin specific sequences did not pass this screening and were thus not included in the downstream analysis. This may explain the low prevalence of hordein, secalin and avenin specific T cells found in this study, where only one single DQ2.5-sec-3-specific TCC was found. T cells that recognize the DQ2.5-hor-3a epitope have been reported to be relatively prevalent in patients that consume barley (8). However, we did not find any DQ2.5-hor-3a TCC in our study possibly due to the limited variety of antigen that was used during the initial screening.

It was a surprise that up to one third of the gluten-reactive TCC apparently have unknown epitope specificity. In comparison, Bodd et al. found two TCC with unknown epitope specificity among 15 gluten-reactive in total (10). Decades of epitope hunting has resulted in an extensive list of total 27 HLA-DQ2.5-restricted gluten/secalin/hordein/avenin epitopes (1). Nevertheless, it is clear that there are still yet uncharacterized HLA-DQ2.5-restricted gluten epitopes. In our study, ‘gluten-only’ TCC was found in five of the six subjects, and was absent only in the subject that had the lowest number of gluten-reactive TCC (n=4). Future studies will show whether these TCC respond to a few commonly recognized novel epitopes, or a multitude of private epitopes.

## Data Availability Statement

The original contributions presented in the study are included in the article/**Supplementary Material**. Further inquiries can be directed to the corresponding author.

## Ethics Statement

The studies involving human participants were reviewed and approved by The regional committee for medical research ethics. The patients/participants provided their written informed consent to participate in this study.

## Author Contributions

S-WQ — Study design, data collection and analysis, and writing of paper. SD-K, LE — Data collection. KL — Procurement of materials, LS — Intellectual contribution to direction of study. All authors contributed to the article and approved the submitted version.

## Funding

This work received support from Centre for Immune Regulation (Research Council of Norway, project 179573/V40 through the Centre of Excellence funding scheme and project 233885), and grants from the Stiftelsen Kristian Gerhard Jebsen for Coeliac Disease Research Centre (SKGJ-MED-017).

## Conflict of Interest

The authors declare that the research was conducted in the absence of any commercial or financial relationships that could be construed as a potential conflict of interest.

## Abbreviations

TCC, T-cell clone; SI, stimulatory index; TG2, transglutaminase 2; CPM, counts per minute.
