# Supplementary material for: Frequency of Gluten-Reactive T Cells in Active Celiac Lesions Estimated by Direct Cell Cloning
Source: Front Immunol. 2021 Mar 16;12:646163. doi: 10.3389/fimmu.2021.646163 (PMC8007869; doi:10.3389/fimmu.2021.646163)
Supplement: Supplementary file 1 [file DataSheet_1.pdf]

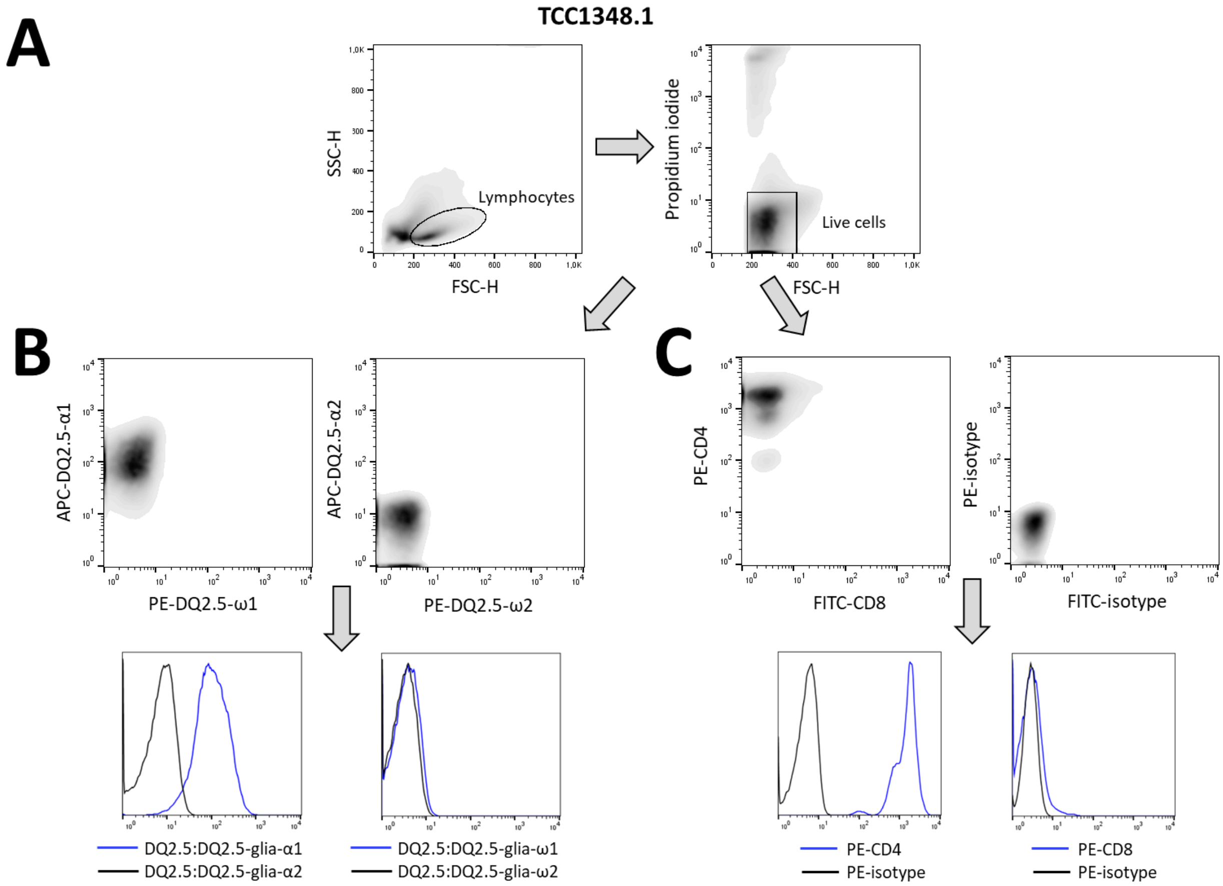

Supplementary Figure 1 Flow cytometric staining of representative T-cell clone (TCC1348.1) with PE-anti-CD4 (SK3), FITC-anti-CD8 (SK1), propidium iodide, and tetramers APC-HLA-DQ2.5:DQ2.5-glia-α1, APC-HLA-DQ2.5:DQ2.5-glia-α2, PE-HLA-DQ2.5:DQ2.5-glia-ω1 and PE-HLA-DQ2.5:DQ2.5-glia-ω2. Live lymphocytes (A) were gated and analysed for either tetramer staining (B) or CD4 / CD8 staining (C).

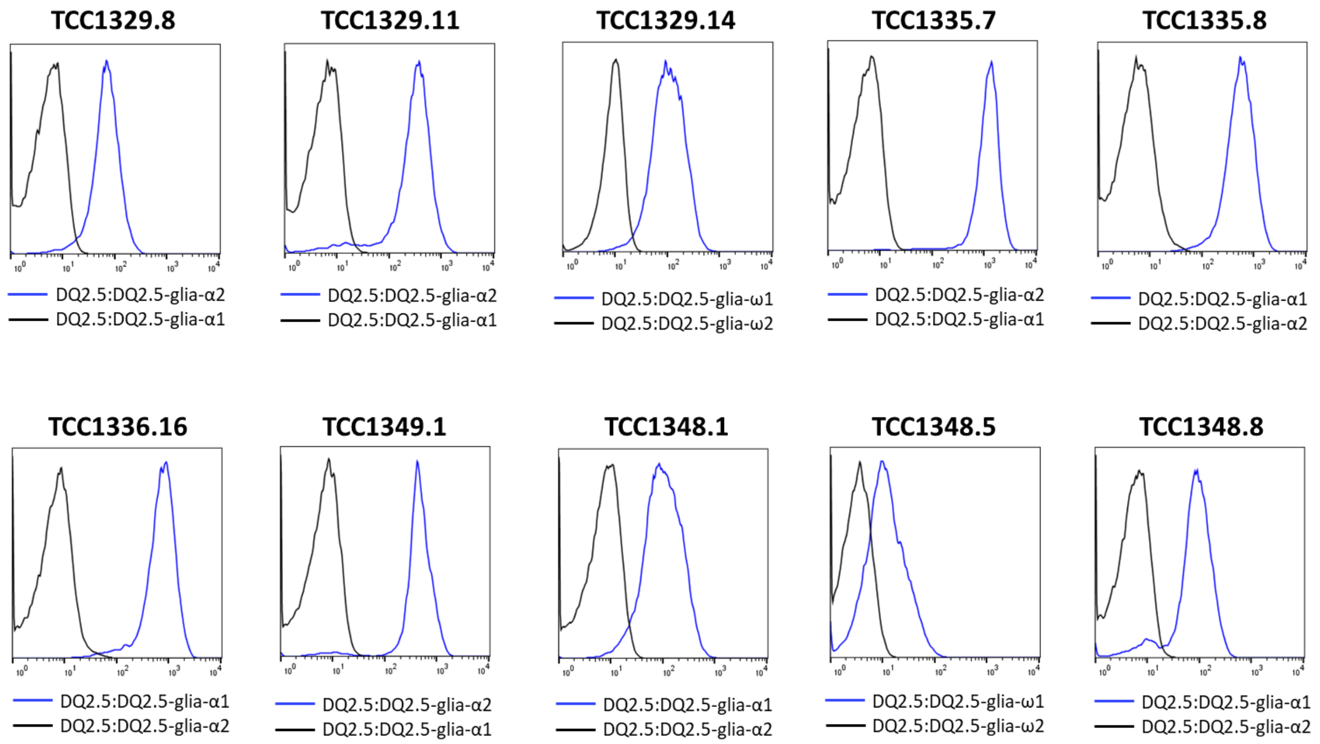

Supplementary Figure 2 Tetramer staining of selected T-cell clones that were reactive to DQ2.5-glia-α1, DQ2.5-glia-α2, DQ2.5-glia-ω1 or DQ2.5-glia-ω2. Each clone was stained with 10 ng/ml each of PE-conjugated HLA-DQ2.5 tetramers presenting either DQ2.5-glia-ω1 or DQ2.5-glia-ω2, and APC-conjugated HLA-DQ2.5 tetramers presenting DQ2.5-glia-α1 or DQ2.5-glia-α2. Live lymphocytes were gated and shown.
